# Supplementary material for: Context-Seq: CRISPR-Cas9 Targeted Nanopore Sequencing for Transmission Dynamics of Antimicrobial Resistance
Source: bioRxiv. 2025 May 31:2024.09.12.612745. Originally published 2024 Sep 12. Preprint. [Version 2] doi: 10.1101/2024.09.12.612745 (PMC11419053; doi:10.1101/2024.09.12.612745)
Supplement: Supplement 1 [file media-1.pdf]

Context-Seq: CRISPR-Cas9 Targeted Nanopore Sequencing for Transmission Dynamics of Antimicrobial Resistance

Erica R. Fuhrmeister, Sooyeol Kim, Shruteek A. Mairal, Caroline McCormack, Benard Chieng, Jenna M. Swarthout, Abigail Harvey Paulos, Sammy M. Njenga, Amy J. Pickering

Supporting Information

List of Tables **Page**

Supplementary Table 1: Taqman Array Card assays 3
Supplementary Table 2: Samples selected for Context-Seq 4
Supplementary Table 3: Candidate guide RNAs for CTX-M and TEM 5
Supplementary Table 4: CTX-M alleles captured with CTX-M guides used in this study 5
Supplementary Table 5: TEM alleles captured with TEM guides used in this study 6
Supplementary Table 6: MinION flow cell ID numbers and SRA sample numbers 7
Supplementary Table 7: Sequencing summary for samples used in this work 7

List of Figures

Supplementary Figure 1: Percentage of sample positive for ARGs and pathogen genes 4
Supplementary Figure 2: Comparison of TEM guide RNAs for enrichment in model system. 5

Commercial Materials
Powersoil Pro kit (Qiagen, 47016), Taqman array cards (Thermo Fisher Scientific, custom order), TaqPath (Applied Biosystems, A15299), crRNAs (IDT, custom), tracrRNAs (IDT, 1072532), duplex buffer (IDT, 11-01-03-01), CutSmart (NEB, B7204), HiFi Cas9 Nuclease V3 (IDT, 1081060), QuickCIP (NEB, M0525S), Thermolabile Proteinase K (NEB, P8111S), dATP (Invitrogen, 10216018), Taq polymerase (NEB, M0273S), Quick Ligation Kit (NEB, M2200S), Adapter Ligation Kit (ONT, SQK-LSK110) AMpure Beads (Beckman Coulter, A63880).

Experimental Methods
Standard Preparation
E. coli were isolated from the stool of children participating in a 2016-2019 cohort study of enteric infections in Lima, Peru (NIH R01AI108695-01A1).[1] Lactose fermenting and non-fermenting colonies were first isolated on MacConkey agar, then sub-plated on Chromagar ESBL to identify presumptive ESBL-producers. ESBL production was phenotypically confirmed using the double disk diffusion assay. The isolate containing CTX-M-55 and TEM-1 used for validation in this work was isolated from a 6-month-old male. For validation experiments, the isolate was plated on MacConkey agar and incubated at 37°C overnight. An individual colony was selected and cultured in 5 mL of LB overnight at 37°C. Media was pelleted and DNA was extracted using Qiagen's Powersoil Pro kit according to the manufacturer's instructions. DNA was quantified on a Qubit fluorometer.

TaqMan Array Card
Samples were pre-amplified with a pool of primers for all targets. A 10 µL reaction was set up with 2.5 µL of TaqPath MM (4x), 2.5 µL of PreAmp Pool, and 5 µL of DNA. Samples were run according to the following thermal cycling conditions: 2 min at 25°C, 1 min at 95°C, 15 cycles of 15 seconds at 95°C and 2 min at 60°C, followed by 10 min at 99°C. Pre-amplified samples were then diluted 20X in nuclease free water. Samples were amplified in a TaqMan Array Card in 100 µL reaction volume composed of 50 µL Taqman Fast Advanced Master Mix, 30 µL nuclease free water, and 20 µL diluted sample. Samples were run according to the following cycling conditions: 2 min at 50°C, 10 min at 92°C, 40 cycles of 95°C for 1 second and 60°C for 20 seconds.

Supplementary Table 1: qPCR assays used on a Taqman Array Card for 14 antibiotic resistance and 8 pathogen genes.

Target	**Forward primer** (5'-3')	**Reverse primer** (5'-3')	**Probe** (5'-3')	Ref
TEM	CACTATTCTCAGAATGACTTGGT	TGCATAATTCTCTTACTGTCATG	CCAGTCACAGAAAAGCATCTTACGG	[2]
TEM-238S	GCTGGCTGGTTTATTGCTGATA	GCCCCAGTGCTGCAATG	ATCTGGAGCCAGTGAG	[3]
qnrS	TTGCTCAGCMTTTATTWCWGGATGT	CAGCGATTTTCAWACARCTCACA	TATGCCAATATGGAGMGGGT	[3]
sul1	CGTGCTGTCGAACCTTCAAA	CCTTTACAGGAAGGCCAACG	AGAAGGATTTCCGCGACAC	[3]
ermB	CGTACCTTGGATATTCACCG	GTAAACAGTTGACGATATTCTCG	TGCACACTCAAGTCTCGATTCAGC	[3]
mcr-1	GATCGCTGTCGTGCTCTTTG	ACCGCGCCCATGATTAATAG	CGATGCTACTGATCACCACG	[3]
CTX-M-1	CCGTCACGCTGTTRTTAGGA	AATGCCACMCCCAGYCKKCC	CAGCAAAAACTTGCCGRATT	[3]
CTX-M-9	GCTTTATGCGCAGACGARTG	ATCACCGCGATAAAGCACCT	TCGATACCRMAGATAATACGC	[3]
tetA	CGACGGCACAGGCTACATC	CCTGGACAACATTGCTTGCA	TGGCGTTCCCGATCAT	[3]
NDM	ATATCACCGTTGGGATCGAC	TAGTGCTCAGTGTCGGCATC	AAGGACAGCAAGGCCAAGTCG	[3]
OXA-48 like	GCAAAGGAATGGCAAGAAAA	CACAACTACGCCCTGTGATTT	AGTTGGAATGCTCACTTTACTG	[3]
OXA-10	CAGAGAAGTTGGCGAAGTAAGA	ATTCTGGTTGGAAGGCCAG	CCTATGGCAACCAGAATATCAGTGGTGG	This study
SHV 238-240SE-SK	GTTGATCCGYTCCGTGCT	GCTTTGTTATKCGGGCCAAG	CGGAGCTAGCRARC	[3]
SHV	TCCCATGATGAGCACCTTTAAA	TCCTGCTGGCGATAGTGGAT	TGCCGGTGACGAACAGCTGGAG	[3]
C. jejuni	CTTGCGGTCATGATGGACATAC	AGCACCACCCAAACCCTCTTCA	TGCTTGCTGCAAAGTATT	[4]
C. coli	AAACCAAAGCTTATCGTGTGC	AGTCCAGCAATGTGTGCAAT	TAAGCTCCAACTTCATCCG	[4]
S. enteritidis	CTTTCTCAGATTCAGGGAGTATATCA	TGAACTACGTTCGTTCTTCTGGT	ATCAGCCTGTTGTCTGCTCACCAT	[5]
S. enterica	TCGGGCAATTCGTTATTGG	GATAAACTGGACCACGGTGACA	AAGACAACAAAACCCACCGC	[6]
Shigella	CCTTTTCCGCGTTCCTTGA	CGGAATCCGGAGGTATTGC	CGCCTTTCCGATACCGTCTCTGCA	[4]
STEC	CCACATCGGTGTCTGTTATTAACC	GGTCAAAACGCGCCTGATAG	TTGCTGTGGATATACGAGG	[4]
ETEC	TTCCCACCGGATCACCAA	CAACCTTGTGGTGCATGATGA	CTTGGAGAGAAGAACCCT	[4]
EPEC	CATTGATCAGGATTTTTCTGGTGATA	CTCATGCGGAAATAGCCGTTA	ATACTGGCGAGACTATTTCAA	[4]

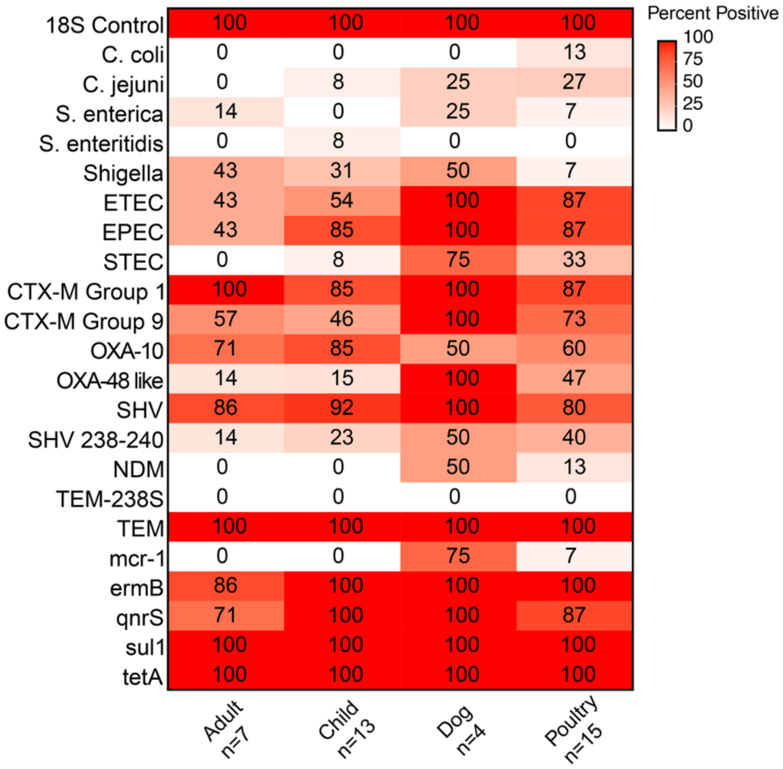

Supplementary Figure 1: Percentage of samples positive for 14 ARGs and 8 pathogen genes using TAC used to pre-screen samples for Context-Seq. Source data are provided in the Source Data file.

Supplementary Table 2: Taqman array card results for TEM and CTX-M in the 4 selected households. 1 indicates a positive detection (CT<40). Sex is indicated for sequenced human samples.

HH	Sample	TEM	CTX-M Group 1	CTX-M Group 9	Selected?	Sex (M/F)
1	Older child	1		1		
1	Child	1	1	1	Y	F
1	Poultry	1	1	1	Y	
1	Dog	1	1	1	Y	
2	Older child	1	1	1		
2	Adult	1	1	1	Y	F
2	Child	1	1			
2	Dog	1	1	1	Y	
2	Poultry 1	1	1	1		
2	Poultry 2	1	1	1		
2	Poultry 3	1	1	1	Y	
4	Adult	1	1	1	Y	F
4	Child	1	1			
4	Poultry 1	1	1	1	Y	
4	Poultry 2	1	1	1		
4	Poultry 3	1				
3	Older child	1	1		Y	M
3	Adult	1	1			
3	Child	1	1		Y	M
3	Poultry 1	1	1	1	Y	
3	Dog	1	1	1	Y	

Supplementary Table 3: Candidate guide RNA screened for use in this study. Mean and standard deviation of off-target hits and off-target scores were calculated by running the custom off-target script on seven human fecal samples from rural Kenya (PRJNA768833).[7]

Guide sequence (5'-3')	Name	Strand	Genomic Location (nt)	Efficiency %	N Off-Target Hits Mean (std)	Off-Target Score Mean (std)
**TEM**						
AGATCAGTTGGGTGCACGAGTGG	BT[1]	+	105	54.47	480 (250)	6 (3)
ACCGCGAGACCCACGCTCACCGG	BR	-	704	51.04	339 (132)	16 (9)
ATACGGGAGGGCTTACCATCTGG	CF[1]	-	745	48.80	346 (97)	33 (8)
ATCGAACTGGATCTCAACAGCGG	AI	+	133	79.59	1308 (240)	9 (6)
**CTX-M**						
TGGCACCACCAACGATATCGCGG	CTX-1	-	729	69.45	2820 (417)	18 (2)
CCAGTTCACGCTGATGGCGACGG	BV[1]	+	21	59.2	3382 (534)	24 (5)
GGTTTTATCCCCCACAACCCAGG	AQ[1]	-	692	58.14	1188 (463)	10 (2)
TTCACTTTTCTTCAGCACCGCGG	CTX-2	-	242	66.44	1019 (305)	13 (3)
AAGTAAGTGACCAGAATCAGCGG	CTX-3	-	775	68.31	448 (71)	21 (6)
GCCGCTGTATGCGCAAACGGCGG	CTX-4	+	72	66.56	1102 (613)	16 (2)

[1]Guide used in this study.

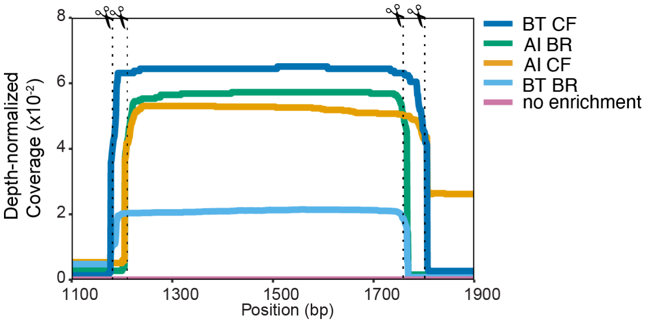

Supplementary Figure 2: Comparison between guide sets in **Supplementary Table 3** for TEM in our test system. Source data are provided in the Source Data file.

Supplementary Table 4: CTX-M alleles captured with CTX-M guides used in this study.

Allele	BV	AQ	Allele	BV	AQ	Allele	BV	AQ
CTX-M-1		1	CTX-M-163	1	1	CTX-M-88	1	1
CTX-M-10	1	1	CTX-M-164		1	CTX-M-96	1	1
CTX-M-12	1	1	CTX-M-166		1	CTX-M-101	1	1
CTX-M-23		1	CTX-M-167	1	1	CTX-M-103	1	1
CTX-M-28		1	CTX-M-169	1	1	CTX-M-114	1	1
CTX-M-29	1	1	CTX-M-170	1	1	CTX-M-116		1
CTX-M-30		1	CTX-M-172	1	1	CTX-M-117	1	1
CTX-M-32		1	CTX-M-173	1	1	CTX-M-123	1	
CTX-M-33	1	1	CTX-M-175		1	CTX-M-132	1	1
CTX-M-34	1	1	CTX-M-176	1	1	CTX-M-136	1	1
CTX-M-36		1	CTX-M-177	1	1	CTX-M-139	1	1
CTX-M-42	1	1	CTX-M-179	1	1	CTX-M-142	1	1
CTX-M-53	1	1	CTX-M-180	1	1	CTX-M-150	1	1
CTX-M-54	1	1	CTX-M-181	1	1	CTX-M-155	1	1
CTX-M-58		1	CTX-M-182	1	1	CTX-M-156	1	1
CTX-M-60	1	1	CTX-M-183	1	1	CTX-M-157	1	1
CTX-M-61		1	CTX-M-184	1	1	CTX-M-158		1
CTX-M-62		1	CTX-M-186	1	1	CTX-M-162	1	1
CTX-M-64	1	1	CTX-M-188	1	1	CTX-M-37	1	1
CTX-M-66	1	1	CTX-M-189	1	1	CTX-M-22	1	1
CTX-M-68	1	1	CTX-M-190	1	1	CTX-M-15	1	1
CTX-M-69	1	1	CTX-M-193	1	1	CTX-M-127	1	1
CTX-M-71	1	1	CTX-M-194	1	1	CTX-M-137		1
CTX-M-72	1	1	CTX-M-197	1	1	CTX-M-138		1
CTX-M-80	1	1	CTX-M-199	1	1	CTX-M-144	1	1
CTX-M-82	1	1	CTX-M-202	1	1	CTX-M-3	1	1
CTX-M-209	1	1	CTX-M-203	1		CTX-M-79	1	1
CTX-M-210	1	1	CTX-M-204	1	1	CTX-M-55	1	1
CTX-M-211	1	1	CTX-M-206		1	CTX-M-52	1	1
CTX-M-212	1	1	CTX-M-207	1	1	CTX-M-218	1	1
CTX-M-216	1	1	CTX-M-208	1	1	CTX-M-220	1	1

Supplementary Table 5: TEM alleles captured with TEM guides used in this study

Allele	BT	CF	Allele	BT	CF	Allele	BT	CF	Allele	BT	CF
TEM-1A	1	1	TEM-86	1	1	TEM-149	1	1	TEM-169	1	1
TEM-1B	1	1	TEM-87	1	1	TEM-150	1	1	TEM-144	1	1
TEM-1C	1	1	TEM-88	1	1	TEM-151	1	1	TEM-129		1
TEM-1D	1	1	TEM-90	1	1	TEM-152	1	1	TEM-128	1	1
TEM-2		1	TEM-91	1	1	TEM-153	1	1	TEM-181	1	1
TEM-2		1	TEM-92	1	1	TEM-154	1	1	TEM-191	1	1
TEM-3		1	TEM-93	1	1	TEM-155		1	TEM-196	1	1
TEM-6	1	1	TEM-94	1	1	TEM-156	1	1	TEM-210	1	1
TEM-8		1	TEM-95	1	1	TEM-157	1	1	TEM-212	1	1
TEM-10	1	1	TEM-96	1	1	TEM-158	1	1	TEM-213	1	1
TEM-11		1	TEM-97	1	1	TEM-159	1	1	TEM-214	1	1
TEM-12	1	1	TEM-98	1	1	TEM-160		1	TEM-215	1	1
TEM-15	1	1	TEM-99	1	1	TEM-162		1	TEM-216		1
TEM-16		1	TEM-101		1	TEM-163	1	1	TEM-217	1	1
TEM-17	1	1	TEM-102	1	1	TEM-164		1	TEM-219	1	1
TEM-20	1	1	TEM-104	1	1	TEM-166	1	1	TEM-220	1	1
TEM-21		1	TEM-105	1	1	TEM-167	1	1	TEM-224	1	1
TEM-22		1	TEM-106	1	1	TEM-168	1	1	TEM-225	1	1
TEM-24		1	TEM-107	1	1	TEM-171	1	1	TEM-226	1	1
TEM-28	1	1	TEM-108	1	1	TEM-176	1	1	TEM-227		1
TEM-29	1	1	TEM-109	1	1	TEM-177		1	TEM-229	1	
TEM-30	1	1	TEM-110	1	1	TEM-178		1	TEM-230	1	1
TEM-33	1	1	TEM-111	1	1	TEM-182	1	1	TEM-231	1	1
TEM-34	1	1	TEM-112	1	1	TEM-183	1	1	TEM-232	1	1
TEM-43	1	1	TEM-113		1	TEM-184	1	1	TEM-233	1	1
TEM-45	1	1	TEM-114		1	TEM-185	1	1	TEM-234	1	1
TEM-47	1	1	TEM-115	1	1	TEM-186	1	1	TEM-148	1	1
TEM-48	1	1	TEM-116	1	1	TEM-187	1	1	TEM-40	1	1
TEM-49	1	1	TEM-120	1	1	TEM-188	1	1	TEM-36	1	1
TEM-52C	1	1	TEM-121		1	TEM-189	1	1	TEM-35	1	1
TEM-52B	1	1	TEM-122	1	1	TEM-190	1	1	TEM-32	1	1
TEM-53	1	1	TEM-123	1	1	TEM-193	1	1	TEM-26	1	1
TEM-54	1	1	TEM-124	1	1	TEM-194	1	1	TEM-19	1	1
TEM-55	1	1	TEM-125	1	1	TEM-195	1	1	TEM-9	1	1
TEM-57	1	1	TEM-126	1	1	TEM-197	1	1	TEM-4	1	1
TEM-60		1	TEM-127	1	1	TEM-198	1	1	TEM-138	1	1
TEM-63	1	1	TEM-130		1	TEM-201	1	1	TEM-139		1
TEM-67		1	TEM-131	1	1	TEM-205	1	1	TEM-141	1	1
TEM-68	1	1	TEM-132	1	1	TEM-206	1	1	TEM-142	1	1
TEM-70	1	1	TEM-133	1	1	TEM-207	1	1	TEM-143	1	1
TEM-71	1	1	TEM-134		1	TEM-208	1	1	TEM-145	1	1
TEM-72		1	TEM-135	1	1	TEM-209		1	TEM-146	1	1
TEM-76	1	1	TEM-136	1	1	TEM-211	1	1	TEM-147	1	1
TEM-77	1	1	TEM-137	1	1	TEM-52	1	1	TEM-84	1	1
TEM-78	1	1	TEM-80	1	1	TEM-82	1	1	TEM-85	1	1
TEM-79	1	1	TEM-81	1	1	TEM-83	1	1			

Supplementary Table 6: MinION flow cell ID numbers and SRA BioSample numbers for BioProject PRJNA1157857.

Sample	Flow Cell Number	SRA BioSample
**HH samples**		
1_child_feces	FAW10902	SAMN43524419
1_poultry_feces	FAW10718<br>FAW05518	SAMN43524420
1_dog_feces	FAW10742	SAMN43524421
2_adult_feces	FAS08862	SAMN43524422
2_dog_feces	FAS96515	SAMN43524423
2_poultry_feces	FAX19523	SAMN43524424
3_child_feces	FAS07765	SAMN43524425
3_older_child_feces	FAS08862	SAMN43524426
3_dog_feces	FAS10189	SAMN43524427
3_poultry_feces	FAS08528	SAMN43524428
4_adult_feces	FAS97950	SAMN43524429
4_poultry1_feces	FAS08896	SAMN43524430
4_poultry2_feces	FAS40845	SAMN43524431
**Optimization**		
regular	FAP82268	SAMN43584091
adaptive	FAQ52471	SAMN43584092
2 guides	FAQ49828	SAMN43584093
2 hr cas9	FAP85007	SAMN43584094
2 targets	FAQ49794	SAMN43584095
no enrichment	FAQ52008	SAMN43584096
ProK	FAS96541	SAMN43584097
Regular enrichment (for proK comparison)	FAS08270	SAMN43584098
CTX-M only	FAS09073	SAMN43584099
TEM only	FAS09238	SAMN43584100
CTX-M and tem together	FAS07765	SAMN43584101
**TEM Guides[1]**		
TEM_AI_BR	AJN275	SAMN43584102
TEM_AI_CF	AJM918	SAMN43584103
TEM_BT_BR	AJN252	SAMN43584104
TEM_CF_BT	AJM933	SAMN43584105
TEM_control	AJN279	SAMN43584106

[1]Flongle flow cell

Supplementary Table 7: Sequencing summary for samples used in this work. Total number of reads, reads containing ARGs, average length of reads, and number of clusters used to generate consensus sequences.

			TEM			CTX-M		
HH	Sample	Total # of reads	# of reads	Ave length	Number of clusters	# of reads	Ave length	Number of clusters
1	Child feces	227,975	79	4314	10	0	-	-
1	Poultry feces	266,102	443	4509	30	0	-	-
1	Dog feces	127,865	856	5886	58	30	3921	6
2	Adult feces	2,669,501	1398	4956	15	0	-	-
2	Dog feces	1,909,047	3235	1818	50	419	1556	10
2	Poultry feces	148,896	2948	4876	116	47	4741	6
3	Child feces	339,046	1778	5268	28	68	5508	5
3	Older child feces	993,679	223	4858	15	3	7106	0
3	Dog Feces	1,915,359	6768	6162	86	0	-	-
3	Poultry feces	199,895	204	4286	27	33	3278	5
4	Adult feces	172,759	59	5751	3	48	4559	4
4	Poultry 1 feces	398,520	341	5385	12	0	-	-
4	Poultry 2 feces	109,002	1128	5037	22	0	-	-

References

1. Pajuelo, M. J. *et al.* Epidemiology of enterotoxigenic Escherichia coli and impact on the growth of children in the first two years of life in Lima, Peru. *Front. Public Health* **12**, (2024).
2. Lachmayr, K. L., Kerkhof, L. J., Dirienzo, A. G., Cavanaugh, C. M. & Ford, T. E. Quantifying nonspecific TEM beta-lactamase (blaTEM) genes in a wastewater stream. *Appl. Environ. Microbiol.* **75**, 203–211 (2009).
3. Pholwat, S. *et al.* Genotypic antimicrobial resistance assays for use on E. coli isolates and stool specimens. *PLOS ONE* **14**, e0216747 (2019).
4. Liu, J. *et al.* Optimization of Quantitative PCR Methods for Enteropathogen Detection. *PLOS ONE* **11**, e0158199 (2016).
5. Kasturi, K. N. & Drgon, T. Real-Time PCR Method for Detection of Salmonella spp. in Environmental Samples. *Appl. Environ. Microbiol.* **83**, e00644-17 (2017).
6. Lappan, R. *et al.* Monitoring of diverse enteric pathogens across environmental and host reservoirs with TaqMan array cards and standard qPCR: a methodological comparison study. *Lancet Planet. Health* **5**, e297–e308 (2021).
7. Fuhrmeister, E. R. *et al.* Evaluating the relationship between community water and sanitation access and the global burden of antibiotic resistance: an ecological study. *Lancet Microbe* **4**, e591–e600 (2023).
