# Supplementary material for: Context-Seq: CRISPR-Cas9 Targeted Nanopore Sequencing for Transmission Dynamics of Antimicrobial Resistance
Source: bioRxiv. 2024 Sep 12:2024.09.12.612745. Preprint. [Version 1] doi: 10.1101/2024.09.12.612745 (PMC11419053; doi:10.1101/2024.09.12.612745)
Supplement: Supplementary file 1 [file media-1.docx]

Context-Seq: CRISPR-Cas9 Targeted Nanopore Sequencing for Transmission Dynamics of Antimicrobial Resistance

Erica R. Fuhrmeister, Sooyeol Kim, Shruteek A. Mairal, Caroline McCormack, Benard Chieng, Jenna M. Swarthout, Abby Paulos, Sammy M. Njenga, Amy J. Pickering

**Supporting Information**

| **List of Tables** | **Page** |
| --- | --- |
| Table S1: Taqman Array Card assays | 3 |
| Table S2: Samples selected for Context-Seq | 4 |
| Table S3: Candidate guide RNAs for CTX-M and TEM | 5 |
| Table S4: CTX-M alleles captured with CTX-M guides used in this study | 5 |
| Table S5: TEM alleles captured with TEM guides used in this study | 6 |
| Table S6: MinION flow cell ID numbers and SRA sample numbers | 7 |
| Table S7: Sequencing summary for samples used in this work | 7 |

| **List of Figures** |  |
| --- | --- |
| Figure S1: Percentage of sample positive for ARGs and pathogen genes | 4 |
| Figure S2: Comparison of TEM guide RNAs for enrichment in model system. | 5 |

**Experimental Methods**

***Standard Preparation***

*E. coli* were isolated from the stool of children participating in a 2016-2019 cohort study of enteric infections in Lima, Peru (NIH R01AI108695-01A1).^1^ Lactose fermenting and non-fermenting colonies were first isolated on MacConkey agar, then sub-plated on Chromagar ESBL to identify presumptive ESBL-producers. ESBL production was phenotypically confirmed using the double disk diffusion assay. The isolate containing CTX-M-55 and TEM-1 used for validation in this work was isolated from a 6-month-old male. For validation experiments, the isolate was plated on MacConkey agar and incubated at 37°C overnight. An individual colony was selected and cultured in 5 mL of LB overnight at 37°C. Media was pelleted and DNA was extracted using Qiagen’s Powersoil Pro kit according to the manufacturer’s instructions. DNA was quantified on a Qubit fluorometer. 

***TaqMan Array Card***

Samples were pre-amplified with a pool of primers for all targets. A 10 μL reaction was set up with 2.5 μL of TaqPath MM (4x), 2.5 μL of PreAmp Pool, and 5 μL of DNA. Samples were run according to the following thermal cycling conditions: 2 min at 25°C, 1 min at 95°C, 15 cycles of 15 seconds at 95°C and 2 min at 60°C, followed by 10 min at 99°C. Pre-amplified samples were then diluted 20X in nuclease free water. Samples were amplified in a TaqMan Array Card in 100 μL reaction volume composed of 50 μL Taqman Fast Advanced Master Mix, 30 μL nuclease free water, and 20 μL diluted sample. Samples were run according to the following cycling conditions: 2 min at 50°C, 10 min at 92°C, 40 cycles of 95°C for 1 second and 60°C for 20 seconds. 

| **Table S1:**qPCR assays used on a Taqman Array Card for 14 antibiotic resistance and 8 pathogen genes. |  |  |  |  |
| --- | --- | --- | --- | --- |
| **Target** | **Forward primer** (5’-3’) | **Reverse primer** (5’-3’) | **Probe** (5’-3’) | **Ref** |
| TEM | CACTATTCTCAGAATGACTTGGT | TGCATAATTCTCTTACTGTCATG | CCAGTCACAGAAAAGCATCTTACGG | ^2^ |
| TEM-238S | GCTGGCTGGTTTATTGCTGATA | GCCCCAGTGCTGCAATG | ATCTGGAGCCAGTGAG | ^3^ |
| qnrS | TTGCTCAGCMTTTATTWCWGGATGT | CAGCGATTTTCAWACARCTCACA | TATGCCAATATGGAGMGGGT | ^3^ |
| sul1 | CGTGCTGTCGAACCTTCAAA | CCTTTACAGGAAGGCCAACG | AGAAGGATTTCCGCGACAC | ^3^ |
| ermB | CGTACCTTGGATATTCACCG | GTAAACAGTTGACGATATTCTCG | TGCACACTCAAGTCTCGATTCAGC | ^3^ |
| mcr-1 | GATCGCTGTCGTGCTCTTTG | ACCGCGCCCATGATTAATAG | CGATGCTACTGATCACCACG | ^3^ |
| CTX-M-1 | CCGTCACGCTGTTRTTAGGA | AATGCCACMCCCAGYCKKCC | CAGCAAAAACTTGCCGRATT | ^3^ |
| CTX-M-9 | GCTTTATGCGCAGACGARTG | ATCACCGCGATAAAGCACCT | TCGATACCRMAGATAATACGC | ^3^ |
| tetA | CGACGGCACAGGCTACATC | CCTGGACAACATTGCTTGCA | TGGCGTTCCCGATCAT | ^3^ |
| NDM | ATATCACCGTTGGGATCGAC | TAGTGCTCAGTGTCGGCATC | AAGGACAGCAAGGCCAAGTCG | ^3^ |
| OXA-48 like | GCAAAGGAATGGCAAGAAAA | CACAACTACGCCCTGTGATTT | AGTTGGAATGCTCACTTTACTG | ^3^ |
| OXA-10 | CAGAGAAGTTGGCGAAGTAAGA | ATTCTGGTTGGAAGGCCAG | CCTATGGCAACCAGAATATCAGTGGTGG | This study |
| SHV 238-240SE-SK | GTTGATCCGYTCCGTGCT | GCTTTGTTATKCGGGCCAAG | CGGAGCTAGCRARC | ^3^ |
| SHV | TCCCATGATGAGCACCTTTAAA | TCCTGCTGGCGATAGTGGAT | TGCCGGTGACGAACAGCTGGAG | ^3^ |
| C. jejuni | CTTGCGGTCATGATGGACATAC | AGCACCACCCAAACCCTCTTCA | TGCTTGCTGCAAAGTATT | ^4^ |
| C. coli | AAACCAAAGCTTATCGTGTGC | AGTCCAGCAATGTGTGCAAT | TAAGCTCCAACTTCATCCG | ^4^ |
| S. enteritidis | CTTTCTCAGATTCAGGGAGTATATCA | TGAACTACGTTCGTTCTTCTGGT | ATCAGCCTGTTGTCTGCTCACCAT | ^5^ |
| S. enterica | TCGGGCAATTCGTTATTGG | GATAAACTGGACCACGGTGACA | AAGACAACAAAACCCACCGC | ^6^ |
| Shigella | CCTTTTCCGCGTTCCTTGA | CGGAATCCGGAGGTATTGC | CGCCTTTCCGATACCGTCTCTGCA | ^4^ |
| STEC | CCACATCGGTGTCTGTTATTAACC | GGTCAAAACGCGCCTGATAG | TTGCTGTGGATATACGAGG | ^4^ |
| ETEC | TTCCCACCGGATCACCAA | CAACCTTGTGGTGCATGATGA | CTTGGAGAGAAGAACCCT | ^4^ |
| EPEC | CATTGATCAGGATTTTTCTGGTGATA | CTCATGCGGAAATAGCCGTTA | ATACTGGCGAGACTATTTCAA | ^4^ |

**Figure S1**: Percentage of samples positive for 14 ARGs and 8 pathogen genes using TAC used to pre-screen samples for Context-Seq. 

| **Table S2:** Taqman array card results for TEM and CTX-M in the 4 selected households. 1 indicates a positive detection (CT<40). |  |  |  |  |  |
| --- | --- | --- | --- | --- | --- |
| **HH** | **Sample** | **TEM** | **CTX-M Group 1** | **CTX-M Group 9** | **Selected?** |
| 1 | Older child | 1 |  | 1 |  |
| 1 | Child | 1 | 1 | 1 | Y |
| 1 | Poultry | 1 | 1 | 1 | Y |
| 1 | Dog | 1 | 1 | 1 | Y |
| 2 | Older child | 1 | 1 | 1 |  |
| 2 | Adult | 1 | 1 | 1 | Y |
| 2 | Child | 1 | 1 |  |  |
| 2 | Dog | 1 | 1 | 1 | Y |
| 2 | Poultry 1 | 1 | 1 | 1 |  |
| 2 | Poultry 2 | 1 | 1 | 1 |  |
| 2 | Poultry 3 | 1 | 1 | 1 | Y |
| 4 | Adult | 1 | 1 | 1 | Y |
| 4 | Child | 1 | 1 |  |  |
| 4 | Poultry 1 | 1 | 1 | 1 | Y |
| 4 | Poultry 2 | 1 | 1 | 1 |  |
| 4 | Poultry 3 | 1 |  |  |  |
| 3 | Older child | 1 | 1 |  | Y |
| 3 | Adult | 1 | 1 |  |  |
| 3 | Child | 1 | 1 |  | Y |
| 3 | Poultry 1 | 1 | 1 | 1 | Y |
| 3 | Dog | 1 | 1 | 1 | Y |

| **Table S3**: Candidate guide RNA screened for use in this study. Mean and standard deviation of off-target hits and off-target scores were calculated by running the custom off-target script on seven human fecal samples from rural Kenya (PRJNA768833).^7^ |  |  |  |  |  |  |
| --- | --- | --- | --- | --- | --- | --- |
| **Guide sequence (5’-3’)** | **Name** | **Strand** | **Genomic Location (nt)** | **Efficiency %** | **N Off-Target Hits Mean (std)** | **Off-Target Score Mean (std)** |
| **TEM** |  |  |  |  |  |  |
| AGATCAGTTGGGTGCACGAGTGG | BT* | + | 105 | 54.47 | 480 (250) | 6 (3) |
| ACCGCGAGACCCACGCTCACCGG | BR | - | 704 | 51.04 | 339 (132) | 16 (9) |
| ATACGGGAGGGCTTACCATCTGG | CF* | - | 745 | 48.80 | 346 (97) | 33 (8) |
| ATCGAACTGGATCTCAACAGCGG | AI | + | 133 | 79.59 | 1308 (240) | 9 (6) |
| **CTX-M** |  |  |  |  |  |  |
| TGGCACCACCAACGATATCGCGG | CTX-1 | - | 729 | 69.45 | 2820 (417) | 18 (2) |
| CCAGTTCACGCTGATGGCGACGG | BV* | + | 21 | 59.2 | 3382 (534) | 24 (5) |
| GGTTTTATCCCCCACAACCCAGG | AQ* | - | 692 | 58.14 | 1188 (463) | 10 (2) |
| TTCACTTTTCTTCAGCACCGCGG | CTX-2 | - | 242 | 66.44 | 1019 (305) | 13 (3) |
| AAGTAAGTGACCAGAATCAGCGG | CTX-3 | - | 775 | 68.31 | 448 (71) | 21 (6) |
| GCCGCTGTATGCGCAAACGGCGG | CTX-4 | + | 72 | 66.56 | 1102 (613) | 16 (2) |

**Figure S2**: Comparison between guide sets in **Table S3** for TEM in our model system. 

| **Table S4**: CTX-M alleles captured with CTX-M guides used in this study. |  |  |  |  |  |  |  |  |
| --- | --- | --- | --- | --- | --- | --- | --- | --- |
| **Allele** | **BV** | **AQ** | **Allele** | **BV** | **AQ** | **Allele** | **BV** | **AQ** |
| CTX-M-1 |  | 1 | CTX-M-163 | 1 | 1 | CTX-M-88 | 1 | 1 |
| CTX-M-10 | 1 | 1 | CTX-M-164 |  | 1 | CTX-M-96 | 1 | 1 |
| CTX-M-12 | 1 | 1 | CTX-M-166 |  | 1 | CTX-M-101 | 1 | 1 |
| CTX-M-23 |  | 1 | CTX-M-167 | 1 | 1 | CTX-M-103 | 1 | 1 |
| CTX-M-28 |  | 1 | CTX-M-169 | 1 | 1 | CTX-M-114 | 1 | 1 |
| CTX-M-29 | 1 | 1 | CTX-M-170 | 1 | 1 | CTX-M-116 |  | 1 |
| CTX-M-30 |  | 1 | CTX-M-172 | 1 | 1 | CTX-M-117 | 1 | 1 |
| CTX-M-32 |  | 1 | CTX-M-173 | 1 | 1 | CTX-M-123 | 1 |  |
| CTX-M-33 | 1 | 1 | CTX-M-175 |  | 1 | CTX-M-132 | 1 | 1 |
| CTX-M-34 | 1 | 1 | CTX-M-176 | 1 | 1 | CTX-M-136 | 1 | 1 |
| CTX-M-36 |  | 1 | CTX-M-177 | 1 | 1 | CTX-M-139 | 1 | 1 |
| CTX-M-42 | 1 | 1 | CTX-M-179 | 1 | 1 | CTX-M-142 | 1 | 1 |
| CTX-M-53 | 1 | 1 | CTX-M-180 | 1 | 1 | CTX-M-150 | 1 | 1 |
| CTX-M-54 | 1 | 1 | CTX-M-181 | 1 | 1 | CTX-M-155 | 1 | 1 |
| CTX-M-58 |  | 1 | CTX-M-182 | 1 | 1 | CTX-M-156 | 1 | 1 |
| CTX-M-60 | 1 | 1 | CTX-M-183 | 1 | 1 | CTX-M-157 | 1 | 1 |
| CTX-M-61 |  | 1 | CTX-M-184 | 1 | 1 | CTX-M-158 |  | 1 |
| CTX-M-62 |  | 1 | CTX-M-186 | 1 | 1 | CTX-M-162 | 1 | 1 |
| CTX-M-64 | 1 | 1 | CTX-M-188 | 1 | 1 | CTX-M-37 | 1 | 1 |
| CTX-M-66 | 1 | 1 | CTX-M-189 | 1 | 1 | CTX-M-22 | 1 | 1 |
| CTX-M-68 | 1 | 1 | CTX-M-190 | 1 | 1 | CTX-M-15 | 1 | 1 |
| CTX-M-69 | 1 | 1 | CTX-M-193 | 1 | 1 | CTX-M-127 | 1 | 1 |
| CTX-M-71 | 1 | 1 | CTX-M-194 | 1 | 1 | CTX-M-137 |  | 1 |
| CTX-M-72 | 1 | 1 | CTX-M-197 | 1 | 1 | CTX-M-138 |  | 1 |
| CTX-M-80 | 1 | 1 | CTX-M-199 | 1 | 1 | CTX-M-144 | 1 | 1 |
| CTX-M-82 | 1 | 1 | CTX-M-202 | 1 | 1 | CTX-M-3 | 1 | 1 |
| CTX-M-209 | 1 | 1 | CTX-M-203 | 1 |  | CTX-M-79 | 1 | 1 |
| CTX-M-210 | 1 | 1 | CTX-M-204 | 1 | 1 | CTX-M-55 | 1 | 1 |
| CTX-M-211 | 1 | 1 | CTX-M-206 |  | 1 | CTX-M-52 | 1 | 1 |
| CTX-M-212 | 1 | 1 | CTX-M-207 | 1 | 1 | CTX-M-218 | 1 | 1 |
| CTX-M-216 | 1 | 1 | CTX-M-208 | 1 | 1 | CTX-M-220 | 1 | 1 |

| **Table S5**: TEM alleles captured with TEM guides used in this study |  |  |  |  |  |  |  |  |  |  |  |
| --- | --- | --- | --- | --- | --- | --- | --- | --- | --- | --- | --- |
| **Allele** | **BT** | **CF** | **Allele** | **BT** | **CF** | **Allele** | **BT** | **CF** | **Allele** | **BT** | **CF** |
| TEM-1A | 1 | 1 | TEM-86 | 1 | 1 | TEM-149 | 1 | 1 | TEM-169 | 1 | 1 |
| TEM-1B | 1 | 1 | TEM-87 | 1 | 1 | TEM-150 | 1 | 1 | TEM-144 | 1 | 1 |
| TEM-1C | 1 | 1 | TEM-88 | 1 | 1 | TEM-151 | 1 | 1 | TEM-129 |  | 1 |
| TEM-1D | 1 | 1 | TEM-90 | 1 | 1 | TEM-152 | 1 | 1 | TEM-128 | 1 | 1 |
| TEM-2 |  | 1 | TEM-91 | 1 | 1 | TEM-153 | 1 | 1 | TEM-181 | 1 | 1 |
| TEM-2 |  | 1 | TEM-92 | 1 | 1 | TEM-154 | 1 | 1 | TEM-191 | 1 | 1 |
| TEM-3 |  | 1 | TEM-93 | 1 | 1 | TEM-155 |  | 1 | TEM-196 | 1 | 1 |
| TEM-6 | 1 | 1 | TEM-94 | 1 | 1 | TEM-156 | 1 | 1 | TEM-210 | 1 | 1 |
| TEM-8 |  | 1 | TEM-95 | 1 | 1 | TEM-157 | 1 | 1 | TEM-212 | 1 | 1 |
| TEM-10 | 1 | 1 | TEM-96 | 1 | 1 | TEM-158 | 1 | 1 | TEM-213 | 1 | 1 |
| TEM-11 |  | 1 | TEM-97 | 1 | 1 | TEM-159 | 1 | 1 | TEM-214 | 1 | 1 |
| TEM-12 | 1 | 1 | TEM-98 | 1 | 1 | TEM-160 |  | 1 | TEM-215 | 1 | 1 |
| TEM-15 | 1 | 1 | TEM-99 | 1 | 1 | TEM-162 |  | 1 | TEM-216 |  | 1 |
| TEM-16 |  | 1 | TEM-101 |  | 1 | TEM-163 | 1 | 1 | TEM-217 | 1 | 1 |
| TEM-17 | 1 | 1 | TEM-102 | 1 | 1 | TEM-164 |  | 1 | TEM-219 | 1 | 1 |
| TEM-20 | 1 | 1 | TEM-104 | 1 | 1 | TEM-166 | 1 | 1 | TEM-220 | 1 | 1 |
| TEM-21 |  | 1 | TEM-105 | 1 | 1 | TEM-167 | 1 | 1 | TEM-224 | 1 | 1 |
| TEM-22 |  | 1 | TEM-106 | 1 | 1 | TEM-168 | 1 | 1 | TEM-225 | 1 | 1 |
| TEM-24 |  | 1 | TEM-107 | 1 | 1 | TEM-171 | 1 | 1 | TEM-226 | 1 | 1 |
| TEM-28 | 1 | 1 | TEM-108 | 1 | 1 | TEM-176 | 1 | 1 | TEM-227 |  | 1 |
| TEM-29 | 1 | 1 | TEM-109 | 1 | 1 | TEM-177 |  | 1 | TEM-229 | 1 |  |
| TEM-30 | 1 | 1 | TEM-110 | 1 | 1 | TEM-178 |  | 1 | TEM-230 | 1 | 1 |
| TEM-33 | 1 | 1 | TEM-111 | 1 | 1 | TEM-182 | 1 | 1 | TEM-231 | 1 | 1 |
| TEM-34 | 1 | 1 | TEM-112 | 1 | 1 | TEM-183 | 1 | 1 | TEM-232 | 1 | 1 |
| TEM-43 | 1 | 1 | TEM-113 |  | 1 | TEM-184 | 1 | 1 | TEM-233 | 1 | 1 |
| TEM-45 | 1 | 1 | TEM-114 |  | 1 | TEM-185 | 1 | 1 | TEM-234 | 1 | 1 |
| TEM-47 | 1 | 1 | TEM-115 | 1 | 1 | TEM-186 | 1 | 1 | TEM-148 | 1 | 1 |
| TEM-48 | 1 | 1 | TEM-116 | 1 | 1 | TEM-187 | 1 | 1 | TEM-40 | 1 | 1 |
| TEM-49 | 1 | 1 | TEM-120 | 1 | 1 | TEM-188 | 1 | 1 | TEM-36 | 1 | 1 |
| TEM-52C | 1 | 1 | TEM-121 |  | 1 | TEM-189 | 1 | 1 | TEM-35 | 1 | 1 |
| TEM-52B | 1 | 1 | TEM-122 | 1 | 1 | TEM-190 | 1 | 1 | TEM-32 | 1 | 1 |
| TEM-53 | 1 | 1 | TEM-123 | 1 | 1 | TEM-193 | 1 | 1 | TEM-26 | 1 | 1 |
| TEM-54 | 1 | 1 | TEM-124 | 1 | 1 | TEM-194 | 1 | 1 | TEM-19 | 1 | 1 |
| TEM-55 | 1 | 1 | TEM-125 | 1 | 1 | TEM-195 | 1 | 1 | TEM-9 | 1 | 1 |
| TEM-57 | 1 | 1 | TEM-126 | 1 | 1 | TEM-197 | 1 | 1 | TEM-4 | 1 | 1 |
| TEM-60 |  | 1 | TEM-127 | 1 | 1 | TEM-198 | 1 | 1 | TEM-138 | 1 | 1 |
| TEM-63 | 1 | 1 | TEM-130 |  | 1 | TEM-201 | 1 | 1 | TEM-139 |  | 1 |
| TEM-67 |  | 1 | TEM-131 | 1 | 1 | TEM-205 | 1 | 1 | TEM-141 | 1 | 1 |
| TEM-68 | 1 | 1 | TEM-132 | 1 | 1 | TEM-206 | 1 | 1 | TEM-142 | 1 | 1 |
| TEM-70 | 1 | 1 | TEM-133 | 1 | 1 | TEM-207 | 1 | 1 | TEM-143 | 1 | 1 |
| TEM-71 | 1 | 1 | TEM-134 |  | 1 | TEM-208 | 1 | 1 | TEM-145 | 1 | 1 |
| TEM-72 |  | 1 | TEM-135 | 1 | 1 | TEM-209 |  | 1 | TEM-146 | 1 | 1 |
| TEM-76 | 1 | 1 | TEM-136 | 1 | 1 | TEM-211 | 1 | 1 | TEM-147 | 1 | 1 |
| TEM-77 | 1 | 1 | TEM-137 | 1 | 1 | TEM-52 | 1 | 1 | TEM-84 | 1 | 1 |
| TEM-78 | 1 | 1 | TEM-80 | 1 | 1 | TEM-82 | 1 | 1 | TEM-85 | 1 | 1 |
| TEM-79 | 1 | 1 | TEM-81 | 1 | 1 | TEM-83 | 1 | 1 |  |  |  |

| **Table S6:** MinION **f**low cell ID numbers and SRA BioSample numbers for BioProject PRJNA1157857. |  |  |  |
| --- | --- | --- | --- |
| **Sample** | **Flow Cell Number** | **SRA BioSample** |  |
| **HH samples** |  |  |  |
| 1_child_feces | FAW10902 | SAMN43524419 |  |
| 1_poultry_feces | FAW10718 | SAMN43524420 |  |
|  | FAW05518 |  |  |
| 1_dog_feces | FAW10742 | SAMN43524421 |  |
| 2_adult_feces | FAS08862 | SAMN43524422 |  |
| 2_dog_feces | FAS96515 | SAMN43524423 |  |
| 2_poultry_feces | FAX19523 | SAMN43524424 |  |
| 3_child_feces | FAS07765 | SAMN43524425 |  |
| 3_older_child_feces | FAS08862 | SAMN43524426 |  |
| 3_dog_feces | FAS10189 | SAMN43524427 |  |
| 3_poultry_feces | FAS08528 | SAMN43524428 |  |
| 4_adult_feces | FAS97950 | SAMN43524429 |  |
| 4_poultry1_feces | FAS08896 | SAMN43524430 |  |
| 4_poultry2_feces | FAS40845 | SAMN43524431 |  |
| **Optimization** |  |  |  |
| regular | FAP82268 | SAMN43584091 |  |
| adaptive | FAQ52471 | SAMN43584092 |  |
| 2 guides | FAQ49828 | SAMN43584093 |  |
| 2 hr cas9 | FAP85007 | SAMN43584094 |  |
| 2 targets | FAQ49794 | SAMN43584095 |  |
| no enrichment | FAQ52008 | SAMN43584096 |  |
| ProK | FAS96541 | SAMN43584097 |  |
| Regular enrichment (for proK comparison) | FAS08270 | SAMN43584098 |  |
| CTX-M only | FAS09073 | SAMN43584099 |  |
| TEM only | FAS09238 | SAMN43584100 |  |
| CTX-M and tem together | FAS07765 | SAMN43584101 |  |
|  | **TEM Guides**^**1**^ |  |  |
| TEM_AI_BR | AJN275 | SAMN43584102 |  |
| TEM_AI_CF | AJM918 | SAMN43584103 |  |
| TEM_BT_BR | AJN252 | SAMN43584104 |  |
| TEM_CF_BT | AJM933 | SAMN43584105 |  |
| TEM_control | AJN279 | SAMN43584106 |  |

^1^Flongle flow cell

| **Table S7:** Sequencing summary for samples used in this work. Total number of reads, reads containing ARGs, average length of reads, and number of clusters used to generate consensus sequences. |  |  |  |  |  |  |  |  |
| --- | --- | --- | --- | --- | --- | --- | --- | --- |
| **HH** | **Sample** | **Total # of reads** | **TEM** |  |  | **CTX-M** |  |  |
|  |  |  | **# of reads** | **Ave length** | **Number of clusters** | **# of reads** | **Ave length** | **Number of clusters** |
| 1 | Child feces | 227,975 | 79 | 4314 | 10 | 0 | - | - |
| 1 | Poultry feces | 266,102 | 443 | 4509 | 30 | 0 | - | - |
| 1 | Dog feces | 127,865 | 856 | 5886 | 58 | 30 | 3921 | 6 |
| 2 | Adult feces | 2,669,501 | 1398 | 4956 | 15 | 0 | - | - |
| 2 | Dog feces | 1,909,047 | 3235 | 1818 | 50 | 419 | 1556 | 10 |
| 2 | Poultry feces | 148,896 | 2948 | 4876 | 116 | 47 | 4741 | 6 |
| 3 | Child feces | 339,046 | 1778 | 5268 | 28 | 68 | 5508 | 5 |
| 3 | Older child feces | 993,679 | 223 | 4858 | 15 | 3 | 7106 | 0 |
| 3 | Dog Feces | 1,915,359 | 6768 | 6162 | 86 | 0 | - | - |
| 3 | Poultry feces | 199,895 | 204 | 4286 | 27 | 33 | 3278 | 5 |
| 4 | Adult feces | 172,759 | 59 | 5751 | 3 | 48 | 4559 | 4 |
| 4 | Poultry 1 feces | 398,520 | 341 | 5385 | 12 | 0 | - | - |
| 4 | Poultry 2 feces | 109,002 | 1128 | 5037 | 22 | 0 | - | - |

**References**

(1)	Pajuelo, M. J.; Noazin, S.; Cabrera, L.; Toledo, A.; Velagic, M.; Arias, L.; Ochoa, M.; Moulton, L. H.; Saito, M.; Gilman, R. H.; Chakraborty, S. Epidemiology of Enterotoxigenic Escherichia Coli and Impact on the Growth of Children in the First Two Years of Life in Lima, Peru. *Front. Public Health* **2024**, *12*. https://doi.org/10.3389/fpubh.2024.1332319.

(2)	Lachmayr, K. L.; Kerkhof, L. J.; Dirienzo, A. G.; Cavanaugh, C. M.; Ford, T. E. Quantifying Nonspecific TEM Beta-Lactamase (blaTEM) Genes in a Wastewater Stream. *Appl. Environ. Microbiol.* **2009**, *75* (1), 203–211. https://doi.org/10.1128/AEM.01254-08.

(3)	Pholwat, S.; Liu, J.; Taniuchi, M.; Chinli, R.; Pongpan, T.; Thaipisutikul, I.; Ratanakorn, P.; Platts-Mills, J. A.; Fleece, M.; Stroup, S.; Gratz, J.; Mduma, E.; Mujaga, B.; Walongo, T.; Nshama, R.; Kimathi, C.; Foongladda, S.; Houpt, E. R. Genotypic Antimicrobial Resistance Assays for Use on E. Coli Isolates and Stool Specimens. *PLOS ONE* **2019**, *14* (5), e0216747. https://doi.org/10.1371/journal.pone.0216747.

(4)	Liu, J.; Gratz, J.; Amour, C.; Nshama, R.; Walongo, T.; Maro, A.; Mduma, E.; Platts-Mills, J.; Boisen, N.; Nataro, J.; Haverstick, D. M.; Kabir, F.; Lertsethtakarn, P.; Silapong, S.; Jeamwattanalert, P.; Bodhidatta, L.; Mason, C.; Begum, S.; Haque, R.; Praharaj, I.; Kang, G.; Houpt, E. R. Optimization of Quantitative PCR Methods for Enteropathogen Detection. *PLOS ONE* **2016**, *11* (6), e0158199. https://doi.org/10.1371/journal.pone.0158199.

(5)	Kasturi, K. N.; Drgon, T. Real-Time PCR Method for Detection of Salmonella Spp. in Environmental Samples. *Appl. Environ. Microbiol.* **2017**, *83* (14), e00644-17. https://doi.org/10.1128/AEM.00644-17.

(6)	Lappan, R.; Henry, R.; Chown, S. L.; Luby, S. P.; Higginson, E. E.; Bata, L.; Jirapanjawat, T.; Schang, C.; Openshaw, J. J.; O’Toole, J.; Lin, A.; Tela, A.; Turagabeci, A.; Wong, T. H. F.; French, M. A.; Brown, R. R.; Leder, K.; Greening, C.; McCarthy, D. Monitoring of Diverse Enteric Pathogens across Environmental and Host Reservoirs with TaqMan Array Cards and Standard qPCR: A Methodological Comparison Study. *Lancet Planet. Health* **2021**, *5* (5), e297–e308. https://doi.org/10.1016/S2542-5196(21)00051-6.

(7)	Fuhrmeister, E. R.; Harvey, A. P.; Nadimpalli, M. L.; Gallandat, K.; Ambelu, A.; Arnold, B. F.; Brown, J.; Cumming, O.; Earl, A. M.; Kang, G.; Kariuki, S.; Levy, K.; Jimenez, C. E. P.; Swarthout, J. M.; Trueba, G.; Tsukayama, P.; Worby, C. J.; Pickering, A. J. Evaluating the Relationship between Community Water and Sanitation Access and the Global Burden of Antibiotic Resistance: An Ecological Study. *Lancet Microbe* **2023**, *4* (8), e591–e600. https://doi.org/10.1016/S2666-5247(23)00137-4.
